# Multiple culprit lesions in ST-segment elevation myocardial infarction with cardiogenic shock: a case of simultaneous thrombosis of two infarct-related arteries

**DOI:** 10.1007/s12471-022-01666-y

**Published:** 2022-02-24

**Authors:** R. Caliskan, H. Ince, F. Arslan

**Affiliations:** 1 Department of Cardiology, Vivantes Klinikum am Urban, Berlin, Germany; 2 Department of Cardiology, Rostock University Medical Centre, Rostock, Germany

**Fig. 1:**
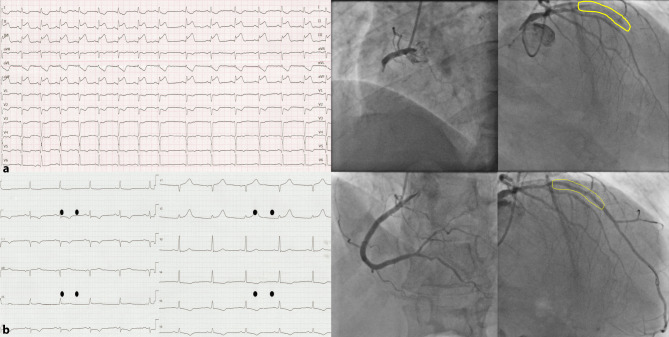
**a**, **b** Electrocardiography (ECG) and angiography of coronary arteries before and after primary percutaneous coronary intervention. **a** ECG at presentation with signs of acute inferior wall myocardial infarction. Diagnostic angiography revealing occlusions of both the proximal right coronary artery (RCA, *left panel*) and left anterior descending artery (LAD) after S1 (*right panel*; lesion site highlighted in *yellow*). **b** Post-procedural ECG showing ST-segment resolution after successful revascularisation of the RCA (*left panel*) and LAD (*right panel*)

A 64-year-old male with previous primary percutaneous coronary intervention (pPCI) of the left anterior descending artery (LAD) presented with cardiogenic shock and inferior wall ST-segment elevation myocardial infarction (STEMI) (Fig. 1a). Coronary angiography revealed an in-stent LAD occlusion with little contrast stasis (Fig. 1a) and stenosis of the circumflex coronary artery (RCx) with poorly perfused collaterals to the right coronary artery (RCA) and LAD. After pPCI of the RCA (Fig. 1b), chest pain persisted without ST-segment resolution. Therefore, we decided to revascularise the LAD on the assumption that it contained fresh thrombus. After easy wiring and pPCI of the LAD, the patient’s complaints and the ST segments resolved with haemodynamic recovery (Fig. 1b). The large, patent first septal branch may have been the reason for the absence of electrocardiographic signs of anterior wall infarction [1]. Multiple culprit vessels are rare in STEMI and associated with cardiogenic shock and high mortality [2, 3]. Further diagnostic studies were negative for hypercoagulability or paradoxical emboli [4]. Being mindful of complaints and electrocardiographic changes may help to guide invasive management of STEMI patients with complicated anatomy.
